# Characterization of an in vitro fed-batch model to obtain cells released from *S. epidermidis* biofilms

**DOI:** 10.1186/s13568-016-0197-9

**Published:** 2016-03-22

**Authors:** Angela França, Virgínia Carvalhais, Manuel Vilanova, Gerald B. Pier, Nuno Cerca

**Affiliations:** CEB-Centre of Biological Engineering, LIBRO-Laboratory of Research in Biofilms Rosário Oliveira, University of Minho, Campus de Gualtar, 4710-057 Braga, Portugal; ICBAS-Instituto de Ciências Biomédicas de Abel Salazar, 4050-313 Porto, Portugal; Instituto de Investigação e Inovação em Saúde, Universidade do Porto, Porto, Portugal; IBMC-Instituto de Biologia Molecular e Celular, Universidade do Porto, Porto, Portugal; Division of Infectious Diseases, Department of Medicine, Brigham and Women’s Hospital, Harvard Medical School, Boston, MA 02115 USA

**Keywords:** Fed-batch systems, Biofilms, Biofilm-released cells, *S. epidermidis*, Antibiotic-tolerance, Gene expression

## Abstract

Both dynamic and fed-batch systems have been used for the study of biofilms. Dynamic systems, whose hallmark is the presence of continuous flow, have been considered the most appropriate for the study of the last stage of the biofilm lifecycle: biofilm disassembly. However, fed-batch is still the most used system in the biofilm research field. Hence, we have used a fed-batch system to collect cells released from *Staphylococcus epidermidis* biofilms, one of the most important etiological agents of medical device-associated biofilm infections. Herein, we showed that using this model it was possible to collect cells released from biofilms formed by 12 different *S. epidermidis* clinical and commensal isolates. In addition, our data indicated that biofilm disassembly occurred by both passive and active mechanisms, although the last occurred to a lesser extent. Moreover, it was observed that *S. epidermidis* biofilm-released cells presented higher tolerance to vancomycin and tetracycline, as well as a particular gene expression phenotype when compared with either biofilm or planktonic cells. Using this model, biofilm-released cells phenotype and their interaction with the host immune system could be studied in more detail, which could help providing significant insights into the pathophysiology of biofilm-related infections.

## Introduction

Biofilms play an essential role in many human infections, including those related to the use of indwelling medical devices (Donlan 2001). Extensive research on biofilms has led to the characterization of the biofilm lifecycle, which encompasses three main stages: (a) initial adhesion, (b) accumulation and (c) dispersion (Otto 2009). Biofilm dispersion is the less studied stage of the biofilm lifecycle. Nevertheless, it is known that the active release of biofilm cells by dispersion is influenced by several environmental cues including temperature and pH fluctuations (Boles and Horswill 2011), accumulation of waste (Kaplan 2010; McDougald et al. 2012), nutrients and oxygen availability (Hunt et al. 2004; Sauer et al. 2004), as well as by the production of signaling molecules (Boles and Horswill 2008; Davies and Marques 2009). In addition to dispersion, biofilms may also release cells into the surrounding environment due to shear forces, and this passive process is often referred as detachment (Choi and Morgenroth 2003; Picioreanu et al. 2001). Biofilm disassembly, which includes both active and passive mechanisms, has been associated with the emergence of serious acute infections (Boles and Horswill 2011). Nevertheless, due to the technical challenges in devising valid and affordable methods to collect cells released from biofilms, the study of these cells has lagged behind. Both fed-batch and dynamic systems have long been used to characterize initial adhesion (Cerca et al. 2005b; Isberg and Barnes 2002), biofilm accumulation and structuring processes (Moormeier and Bayles 2014; Periasamy et al. 2012). However, no fed-batch system has been used so far to study biofilm disassembly. Despite the advantages of dynamic over fed-batch systems, such as tight control of hydrodynamic systems and continuous diffusion of nutrients and waste, it presents important drawbacks such as difficulty in assembling systems, frequent formation of air bubbles that destroy biofilm architecture (Crusz et al. 2012), limited number of conditions that can be analyzed simultaneously and higher cost. Hence, the development of a model to study the cells released from biofilms based on the widely used fed-batch systems may provide significant insights into the pathophysiology of biofilm-related infections.

Owing to its ubiquitous presence in human skin, *Staphylococcus epidermidis* frequently colonizes and form biofilms on skin penetrating devices such as vascular catheters being, thus, considered one of the major nosocomial pathogens associated with biomaterial-associated biofilms infections (Otto 2009). Due to the release of cells from biofilms formed on vascular catheters into the surrounding environment, *S. epidermidis* is responsible for 22 % of bloodstream infections detected in intensive care units patients, in the USA (Otto 2013). It is, therefore, important to study in more detail the phenotype and the interaction of these cells with the host immune system, in order to conceive effective strategies to control the pathologic events associated with biofilm disassembly. Herein, we describe a simple and affordable fed-batch method to collect cells disassembled from *S. epidermidis* biofilms that can be used for further functional studies.

## Materials and methods

### Bacteria and growth conditions

*S. epidermidis* strain 9142 (isolated from blood culture by (Mack et al. 1992) (collection number 18857 at DSM, Braunschweig, Germany) was selected for the main experiments conducted in this study. The clinical and commensal strains used for validation purposes are listed in Table 1. Biofilms were grown, in 24-well plates made of polystyrene plastic (Orange Scientific, Braine-l’Alleud, Belgium), by inoculating 1.5 µL of an overnight culture into 1 mL of Tryptic Soy Broth (Liofilchem, Teramo, Italy) supplemented with glucose to a final concentration of 0.65 % (v/v) (TSB_0.65 %G_), and by incubating at 37 °C with agitation at 120 rpm. After 24 h of growth, and prior to any of the subsequent experimental manipulations, biofilms were washed twice with 0.9 % NaCl solution (ChemLab, West-Vlaaderen, Belgium). Planktonic cultures, both exponential and stationary phase, were started by inoculating 1 × 10^7^ cells/mL bacteria in 10 mL of TSB_0.65 %G_ in a 25 mL Erlenmeyer and by incubating at 37 °C under agitation at 120 rpm for, respectively, 6 and 24 h.

**Table 1:** *Staphylococcus epidermidis* strains used for the validation of glucose influence in biofilm dispersion

Strain	Description	Study
RP62A	ATCC reference strain 35984	Christensen et al. (1985)
1457	Reference strain used in biofilm-related studies	Mack et al. (1994)
IE186	Clinical isolate from a patient with infective endocarditis	Cerca et al. (2006)
IE214	Clinical isolate from a patient with infective endocarditis	Cerca et al. (2006)
PT11006	Clinical isolate from patient after a kidney transplant	a
PT12003	Clinical isolate from a patient after a stomach surgery	a
SECOM005A	Commensal isolates of the human skin	Oliveira and Cerca (2013)
SECOM20A1		
SECOM030A		
SECOM58A		

^a^ Unpublished isolate obtained from an on-going epidemiological study in Portugal. Isolates were obtained after patient informed consent with the approval of ethical committee the Hospital Geral de Santo António, Porto, Portugal. Each isolate was first identified at the species level using the commercially available VITEK® two identification system using the gram-positive ID card (BioMérieux, Marcy l’Etoile, France) and molecular identification was confirmed by sequencing of the *rpoB* gene (Mellmann et al. 2006)

### Influence of different glucose concentrations in biofilm disassembly, under agitation or static conditions

TSB without glucose (TSB_0 %G_) (1 L) was prepared by mixing the following individual components: 17 g pancreatic digest casein (Alfa Aeser, Karlsruhe, Germany), 3 g papaic digest of soybean (Fluka, MO, USA), 5 g NaCl (ChemLab) and 2.5 g dipotassium hydrogen phosphate (ChemLab). In order to test the effect of varying glucose concentrations on biofilm disassembly, TSB_0 %G_ was supplemented with different quantities of glucose to obtain the following final concentrations (v/v): TSB_0.25 %G_, TSB_0.65 %G_ or TSB_1.25 %G_. Each medium was carefully added to 24 h-old biofilms and incubated at 37 °C for additional 24 h, without or with agitation at 120 rpm. The cells present in the biofilm bulk fluid were collected by careful aspiration. Biofilms were washed twice and then suspended in 1 mL of 0.9 % NaCl. Both bacterial cultures were sonicated for 5 s at 40 % amplitude using a 13 mm probe tip (Cole-Parmer 750-Watt Ultrasonic Homogenizer 230 VAC, IL, USA). This sonication cycle did not significantly affect cells viability as demonstrated before (Freitas et al. 2014). Thereafter, biofilms and the cells in the biofilm bulk fluid were quantified regarding their total biomass and the number of total or cultivable bacteria, as described below.

### Quantification of bacterial biomass or bacterial cells

Bacterial biomass was determined by optical density (OD), at 640 nm of wavelength, as previously described (Freitas et al. 2014). The number of cultivable cells was determined by the standard colony forming units (CFU) counting. The number of total cells was assessed by flow cytometry (EC800, SONY, San Jose, CA, USA) using SYBR Green (Invitrogen, Glasgow, UK) and propidium iodide (Sigma, St. Gallen, Switzerland) staining as previously optimized (Cerca et al. 2011b).

### Biofilm-released cells dynamics under static or agitation conditions

After discarding the spent medium and washing twice the preformed 24 h-old biofilms, 1 mL of TSB_0.65 %G_ was carefully added. Immediately after, in half of the wells, the newly added TSB_0.65 %G_, together with the cells released from the biofilm due to the shear forces exerted by medium addition, was transferred into empty sterile wells. The cells in the biofilm bulk fluid were incubated, in the presence or absence of the originating biofilm, at 37 °C with shaking at 120 rpm or under static conditions. At different time points, a 20 µL aliquot was collected from both conditions and sonicated for 2 s at 20 % amplitude. The number of total and cultivable cells was determined, respectively, by flow cytometry and CFU counting. Four independent experiments with three technical replicates were performed.

### Susceptibility of *S. epidermidis* populations to vancomycin, tetracycline and rifampicin

One mL of fresh TSB_0.65 %G_ was added to the preformed 24 h-old biofilms and these were allowed to grow for additional 24 h at 37 °C with agitation at 120 rpm. At time points 0, 12 and 24 h, the bulk fluid of 10 individual biofilms was carefully collected by aspiration and pooled together. For comparative purposes, planktonic cultures obtained after 6 h (exponential) or 24 h of growth (stationary), as well as biofilm cells obtained by disrupting the established biofilms as described elsewhere (Cerca et al. 2011a), were also analysed. Cells from all populations were harvested by centrifugation, suspended in 0.9 % NaCl and sonicated for 5 s at 40 % amplitude. Finally, the number of total cells was quantified by flow cytometry and the concentration adjusted to 1 × 10^7^ cells/mL in TSB_0.25 %G_ with or without antibiotics. Antibiotics (all from Sigma) of three different classes, and with different mechanisms of action, were used at their peak serum concentrations: 40 mg/L of vancomycin (a cell wall synthesis inhibitor), 16 mg/L of tetracycline (a protein synthesis inhibitor) and 10 mg/L rifampicin (DNA-dependent RNA polymerase enzyme inhibitor) (Cerca et al. 2005a). These cells were incubated for 2 h, in 10 mL tubes, at 37 °C and agitation at 120 rpm. Thereafter, bacteria were collected by centrifugation, suspended in 0.9 % NaCl and sonicated for 5 s at 40 % amplitude for posterior CFU counting. This experiment was repeated three independent times with two technical replicates.

### Reversibility of antimicrobial susceptibility phenotype of biofilm-released cells

The concentration of the cells in the biofilm bulk fluid obtained immediately after medium replacement (Brc.0) or after 24 h of incubation in the presence of the originating biofilms (Brc.24), was adjusted to 1 × 10^7^ cells/mL in TSB_0.25 %G_ after quantification by flow cytometry. Both cultures were transferred into 25 mL sterile Erlenmeyer and incubated, up to 4 h, at 37 °C with agitation at 120 rpm. At time points 0, 2 and 4 h, 20 µL aliquots were collected, counted by flow cytometry and their concentration adjusted to 1 × 10^7^ cells/mL in TSB_0.25 %G_ or TSB_0.25 %G_ containing 40 mg/L of vancomycin. The susceptibility to vancomycin was then assessed under the same conditions described above. Three independent experiments were performed with two technical replicates.

### Quantification of gene expression levels in *S. epidermidis* populations

Quantitative PCR (qPCR) analysis was performed, as described elsewhere (Franca et al. 2012), in order to determine the levels of transcription of genes involved in quorum sensing (*agrB*), biofilm accumulation (*icaA*) and dispersion (*atlE*, *psmβ*). In brief, after pooling together the bulk fluid of 10 biofilms, the 10 originating biofilms were washed twice and pooled together as well, in order to reduce the characteristic heterogeneity of this population (Sousa et al. 2014). Thereafter, 2 mL of planktonic (24 h stationary phase), biofilm bulk fluid or biofilm cells were collected by centrifugation and total RNA immediately isolated using FastRNA^®^ Pro blue Kit (MP Biomedicals, Santa Ana, CA, USA). Henceforth, 0.5 µg of total RNA was reverse transcribed into complementary DNA in the presence of the enzyme RevertAid™ Premium reverse transcriptase (Thermo Scientific, Lisbon, Portugal). Random primers (NZYTech, Lisbon, Portugal) were used as priming strategy. The primers used for qPCR experiments were designed using Primer3 software (Rozen and Skaletsky 2000) having *S. epidermidis* RP62A complete genome (PubMed accession number NC_002976.3) or ATCC 12228 (PubMed accession number NC_004461.1) as a template. The size of the amplicon and the sequence of the primers used for the quantification of the transcripts encoding *icaA*, *psmβ*, *agrB* and 16 ribosomal RNA have been previously described (Franca et al. 2012). The sequence of the primers used for the detection of *atlE* transcripts, which yield a 180 base pair amplicon, are: Fw5′-GTAGATGTTGTGCCCCAAGG-3′ and Rv: 5′-TGGAAGAGGAACAGTTTGGAC-3. The qPCR run was performed using CFX96™ thermal cycler (Bio-Rad, Hercules, CA, USA) with the following cycling parameters: 10 min at 95 °C followed by 40 repeats of 5 s at 95 °C, 10 s at 58 °C and 20 s at 72 °C. The qPCR reaction was prepared by mixing 5 µL of iQ™ SYBR Green Supermix (Bio-Rad), 0.5 µL of each forward and reverse primers at 10 µM, 2 µL of ultrapure water and finally, 2 µL of 1:400 diluted cDNA. Melting curves were analyzed for the detection of unspecific products or primer dimer formation. The efficiency of the qPCR reactions (with all set of primers) was calculated using several dilutions of cDNA from biofilm samples. Fold change expression was determined by applying a variation of the Livak and Schmittgen method, the Efficiency^∆Ct^ method, using 16 rRNA as reference gene and biofilm cells as control sample. Data analysis was based on at least four independent experiments.

### Statistical analysis

Graphs and statistical analysis were performed with GraphPad Prism version 6 (CA, USA). Data are depicted as means and standard deviation. Unpaired *T* tests with Welch’s correction or One-way ANOVA with Tukey’s, Dunnett’s or Bonferroni’s multiple comparison tests were used when appropriate (the statistical test used is indicated in each figure legend). For gene expression studies, since a normal distribution of the values determined was not found, data are depicted as medians with interquartile range and statistical differences among populations were determined using Kruskal–Wallis with Dunn’s multiple comparison test. Differences among groups were considered significant when *P* was less than 0.05.

## Results

### Glucose modulates biofilm disassembly in *S. epidermidis,* under agitation or static conditions

**Fig. 1:**
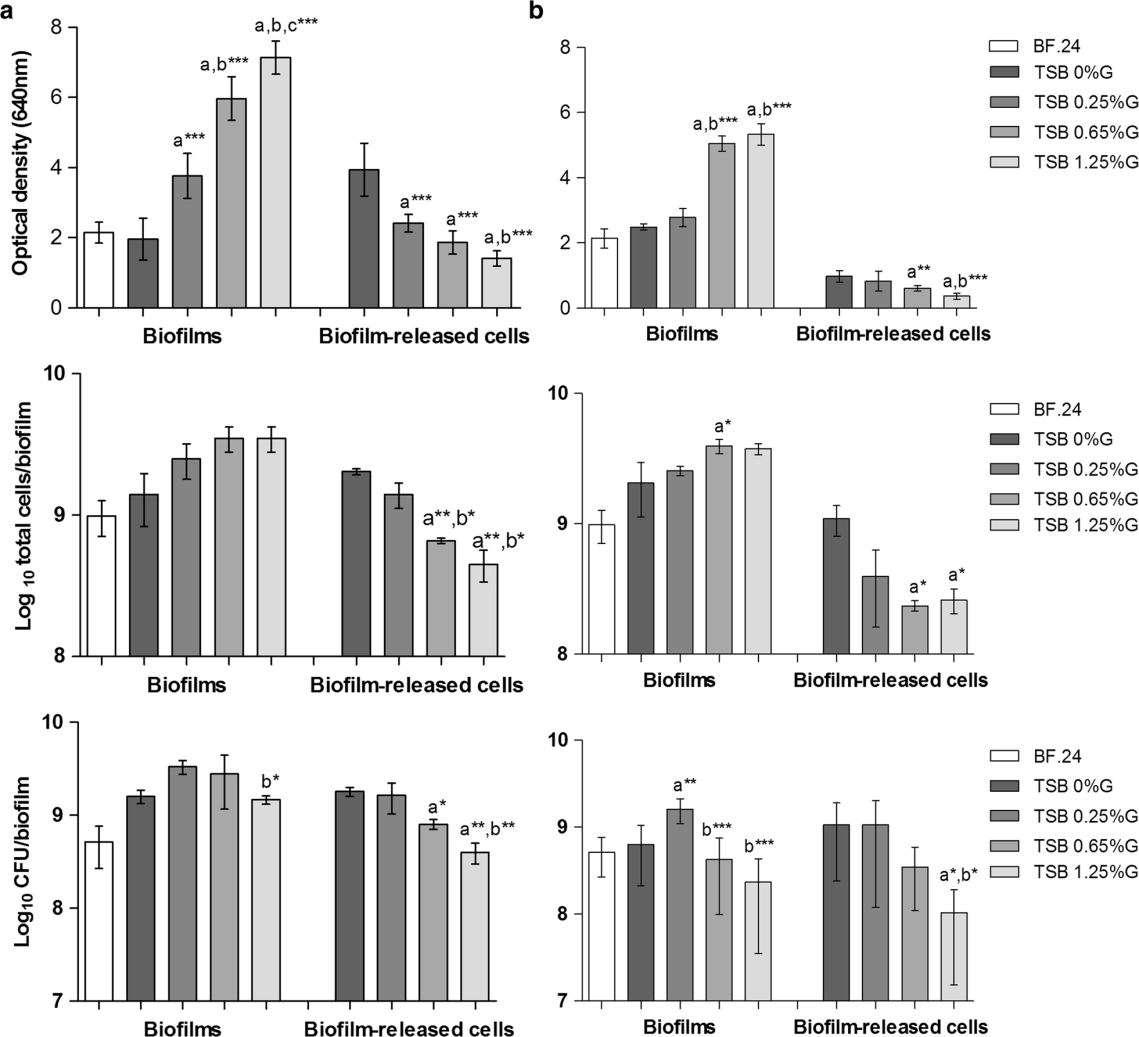
The influence of different glucose concentrations on biofilm disassembly. Twenty-four hour-old biofilms (BF.24) were incubated with TSB without (TSB_0 %G_) or supplemented with 0.25 % (TSB_0.25 %G_), 0.65 % (TSB_0.65 %G_) or 1.25 % (TSB_1.25 %G_) of glucose and allowed to grow for additional 24 h, under agitation (**a**) or static conditions (**b**). The values represent the average ± the standard deviation of three independent experiments. Statistical significance among groups was determined using One-way ANOVA and Tukey’s multiple comparison test. **a** Statistical significance when comparing with TSB_0 %G_, **b** when comparing with TSB_0.25 %G_ and **c** when comparing with TSB_0.65 %G_. *P < 0.05, **P < 0.01, ***P < 0.001. *BF* biofilms

**Fig. 2:**
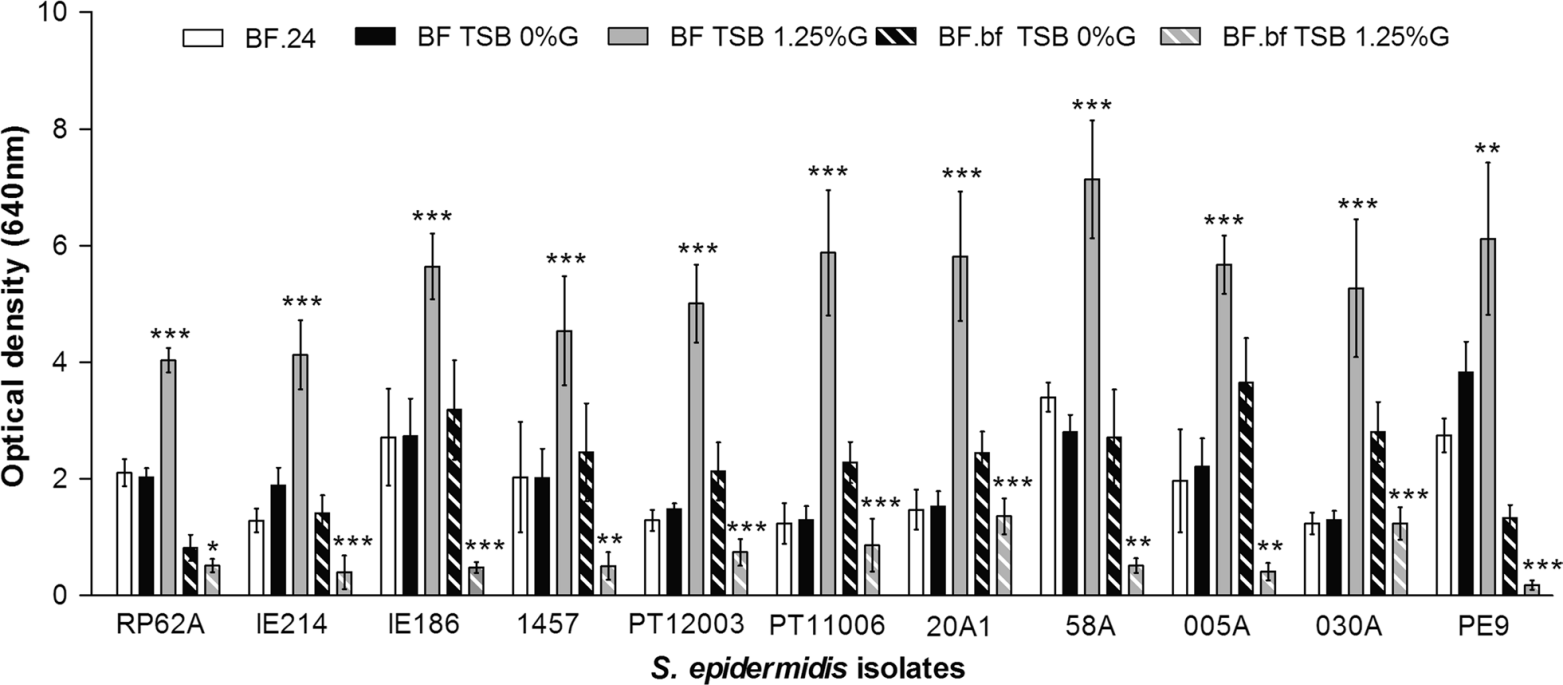
Evaluation of biofilm disassembly in distinct *S. epidermidis* clinical and commensal isolates. Twenty-four hour-old biofilms (BF.24) formed by each of the strains indicated were incubated for additional 24 h, under agitation conditions, with either TSB_0 %G_ or TSB_1.25 %G_. The *bars* represent the average ± standard deviation of two independent experiments. Statistical significance was determined using Unpaired *T* test (Two-tailed) with Welch’s correction. *P < 0.05, **P < 0.01, ***P < 0.001. *BF.bf* biofilm bulk fluid

By analyzing the biomass and the number of total and cultivable cells, we were able to follow biofilm dynamics in different glucose concentrations and under different hydrodynamic conditions (with or without agitation). As expected, biofilm accumulation augmented with increasing concentrations of glucose (Mack et al. 1992), independently of shear forces (Fig. 1). However, this effect was more pronounced in biofilms grown under agitation conditions. On the other hand, the opposite trend was observed when looking at the cells in the biofilm bulk fluid. The ratio of total cells in biofilms/bulk fluid was 69 % in TSG_0 %G_, 179 % in TSB_0.25 %G_, 530 % in TSB_0.65 %G_ and 778 % in TBS_1.25 %G_. When incubating biofilms with TSB_1.25 %G_, a decrease in the number of cultivable cells was observed, being this more evident when growing biofilms without agitation (Fig. 1b). This phenomenon is due to the excess of glucose in the growth medium, a condition that promotes dormancy of the bacterial cells within *S. epidermidis* biofilms (Cerca et al. 2011a). In addition, in order to understand if the effect of glucose was strain independent, we selected 11 previously characterized strains: six clinical and five commensal isolates (Table 1), and quantified biofilms and the cells in the bulk fluid in the two extreme conditions tested: absence (TSB_0 %G_) or presence of excess of glucose (TSB_1.25 %G_). As shown in Fig. 2, the same tendency was observed with all strains tested indicating that the effect of glucose is indeed strain independent.

### The majority of cells present in the biofilm bulk fluid result from both biofilm dispersion and detachment processes

**Fig. 3:**
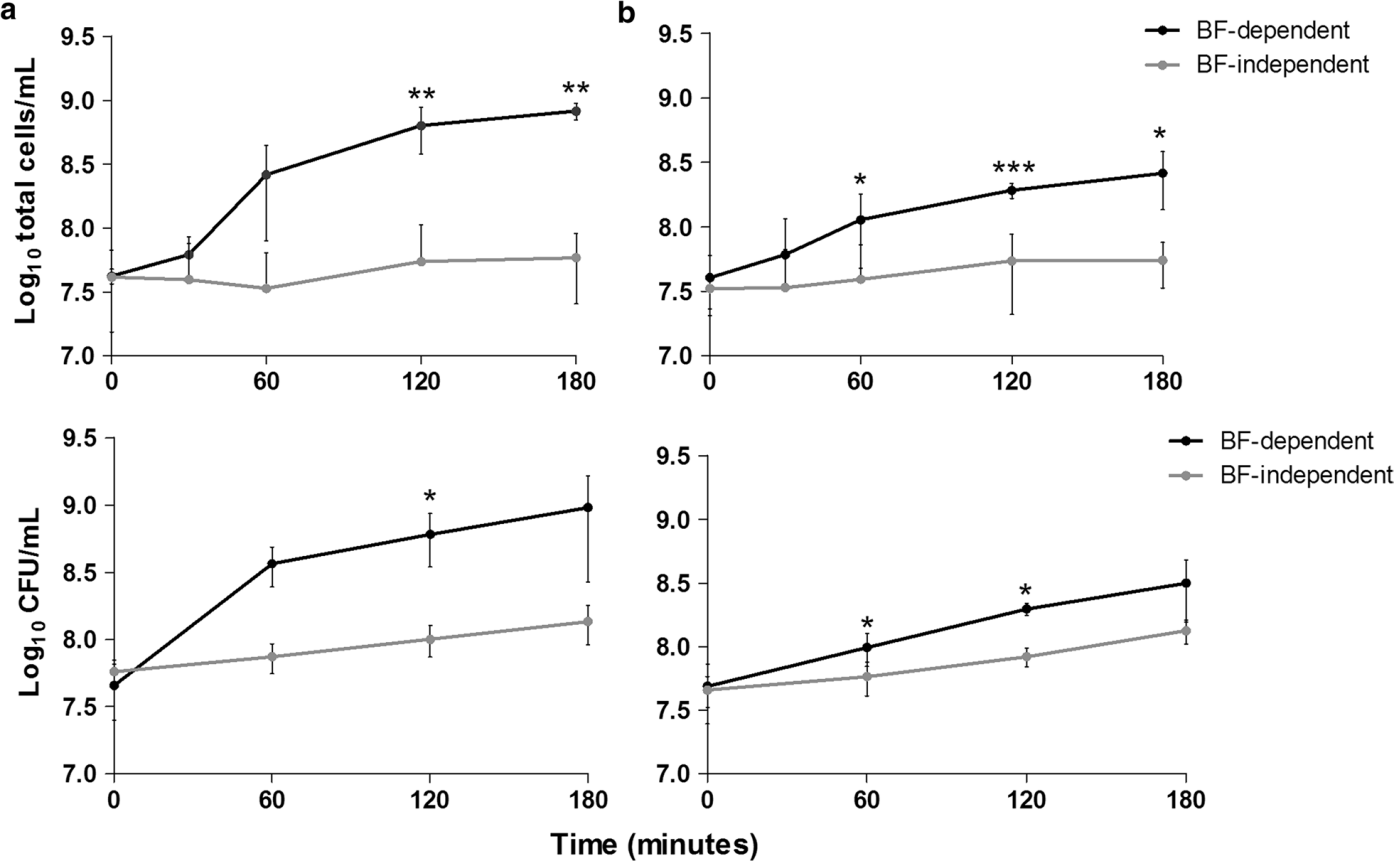
Growth kinetics of Brc in the presence or absence of the originating biofilms, under agitation or static conditions. Twenty-four hours-old biofilms were incubated with TSB_0.65 %G_ for 180 min under agitation (**a**) or static conditions (**b**). Every 60 min an aliquot was collected and the number of total or cultivable cells was determined, respectively, by flow cytometry and by CFU counting. The *dots* represent the average and *error bars* the standard deviation of 2–3 independent experiments. Statistical significance was determined using Unpaired *T* test (Two-tailed) with Welch’s correction. *P < 0.05, **P < 0.01, ***P < 0.001. The section c illustrates the experimental set-up of the assay performed to obtain the results depicted in *panels* **a** and **b**. *BF* biofilms

In order to determine if the cells present in the biofilm bulk fluid resulted from the proliferation of planktonic bacteria carried-over from in the initial 24 h of growth, or from biofilm disassembly (by active or passive mechanisms), fresh TSB_0.65 %G_ was added to 24 h-old biofilms being, in half of the cases, immediately transferred into empty culture plate wells (see Fig. 3c). The cell density in the suspensions grown in the presence or in the absence of the originating biofilms was compared. As shown in Fig. 3a, the bacterial density in wells containing biofilms on the bottom increased more than 20-fold in the 3 h-period tested, while it only increased around 2-fold when growing in the absence of the originating biofilms, which corresponds to the *S. epidermidis* doubling time. When the same experiment was performed without the influence of shear forces (without agitation), a similar trend was observed with a 6-fold increase in cell density in the presence of the originating biofilms and nearly a 2-fold increase in their absence (Fig. 3b). These results demonstrated that the great majority of the cells in the biofilm bulk fluid are being released from the biofilm, by both active and passive mechanisms. Hence, we named the biofilm bulk fluid as biofilm-released cells (Brc).

### Washing and growth medium addition procedures result in biofilm detachment

**Fig. 4:**
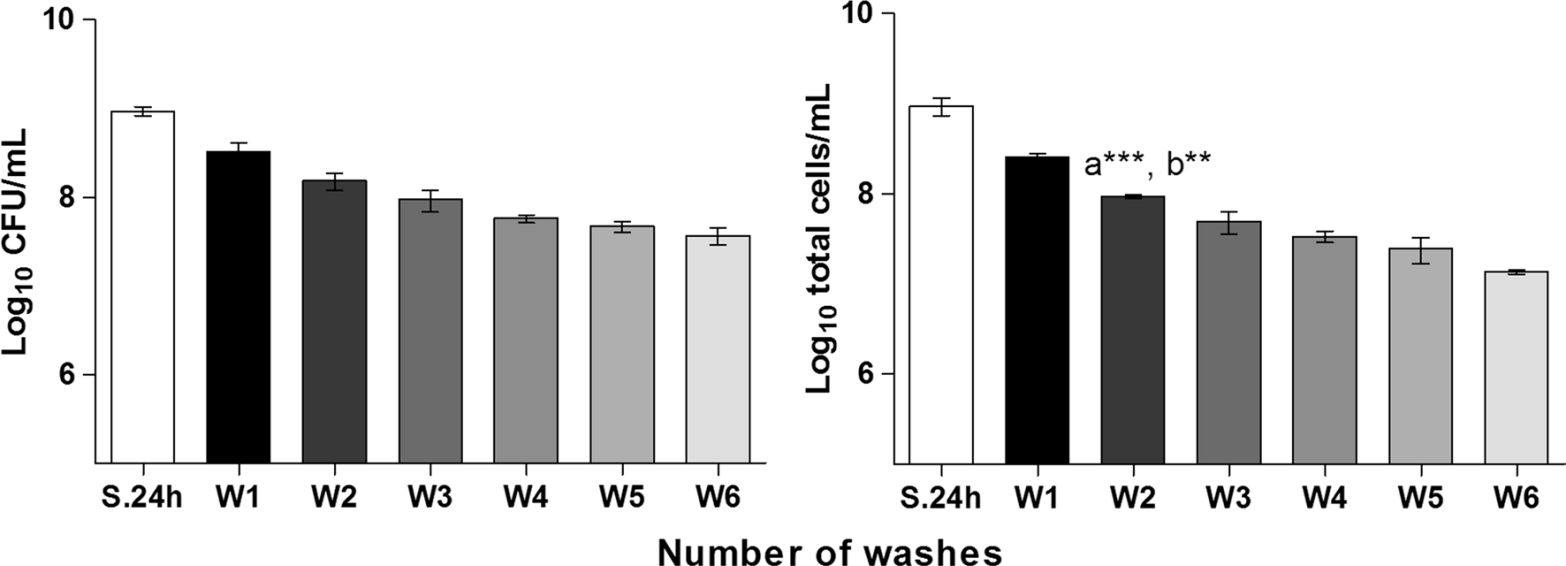
Number of cells removed from the biofilm due to the washing and medium addition procedures. Cultivable and total cell numbers recovered in the saline solution used in each washing step (W) were respectively determined by CFU counting (**a**) and flow cytometry (**b**). The number of cells in the 24 h-old biofilm suspensions, which was discarded before biofilm washing step, is represented as S.24 h (suspension 24 h). *Bars* represent the average ± standard deviation of 2–3 independent experiments. Statistical significance was determined using One-way ANOVA and Bonferroni’s multiple comparison test. **a** Statistical significance when comparing with the previous wash and **b** when comparing with the following wash. **P < 0.01, ***P < 0.001

When using fed-batch systems, the replacement of spent medium and the inherent washing steps are fundamental to replenish nutrients and to remove, respectively, non- or biofilm loosely adherent cells. Nevertheless, this procedure might also result in the detachment of cells from the biofilm due to the shear forces exerted. Hence, in order to verify the influence of both washing and medium addition procedures in the initial quantity of cells in the biofilm bulk fluid, sequential washes were performed in the same biofilm, and the number of total and cultivable bacteria was determined in the saline solution obtained after each wash. A significant difference in the number of total cells recovered was observed between the first and the third washing step, although a tendency to recover decreasing numbers of total and cultivable cells was consistently observed from wash one to wash six (Fig. 4).

### Biofilm-released cells are more tolerant to vancomycin and tetracycline than planktonic cells

**Fig. 5:**
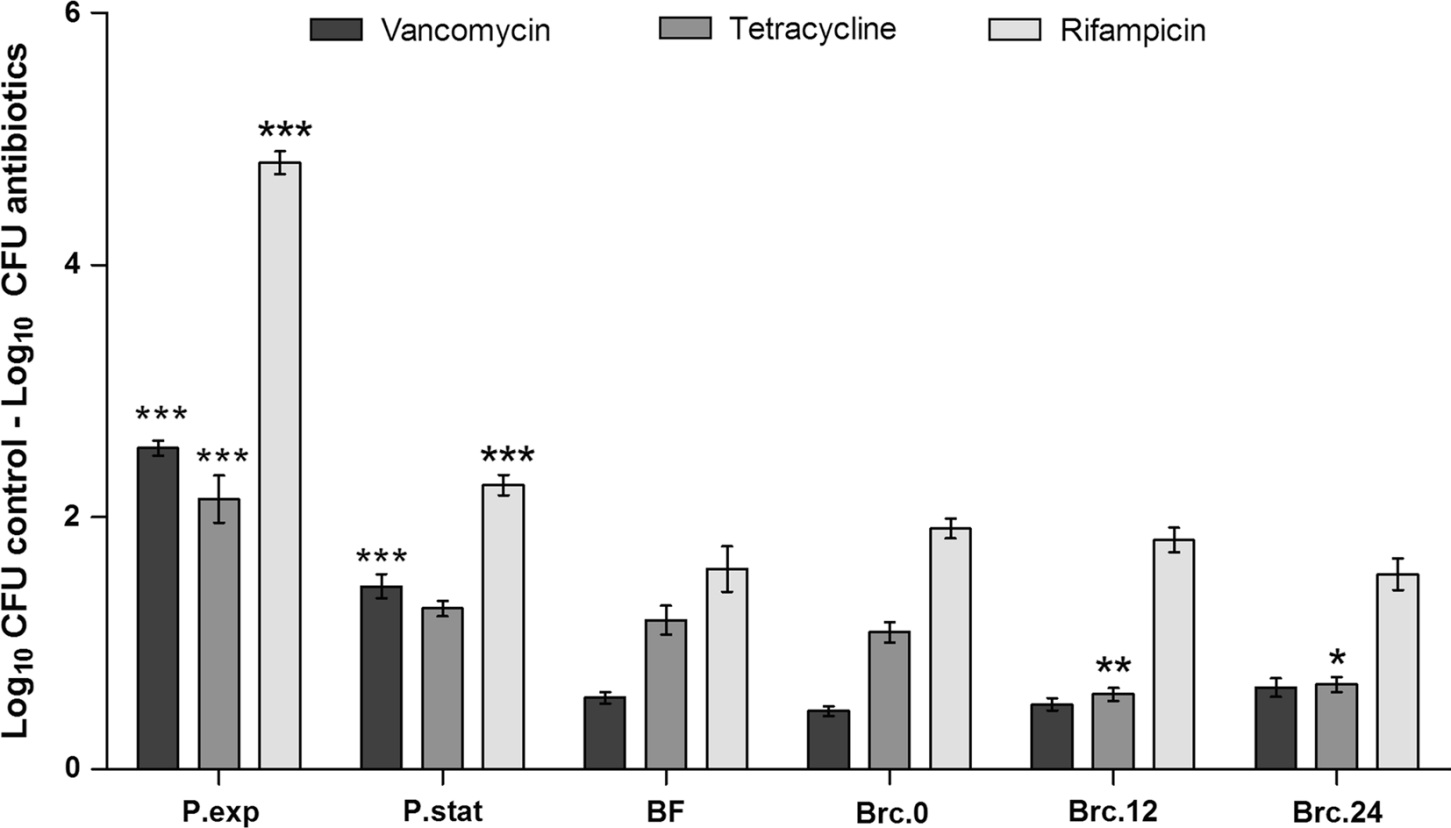
Susceptibility of the different *S. epidermidis* populations to vancomycin, tetracycline and rifampicin. Exponential (exp) or stationary (stat) phase planktonic (P) cells, cells from disrupted biofilms (BF) or Brc were collected at each time point indicated and their concentration adjusted to 1 × 10^7^ cells/mL in TSB_0.25 %G_ or TSB_0.25 %G_ containing each antibiotic and incubated for 2 h at 37 °C and 120 rpm. *Bars* represent the average ± standard deviation of three independent experiments. Statistical significance was determined using One-way ANOVA and Dunnett’s Multiple Comparison test. *P < 0.05, **P < 0.01, ***P < 0.001

**Fig. 6:**
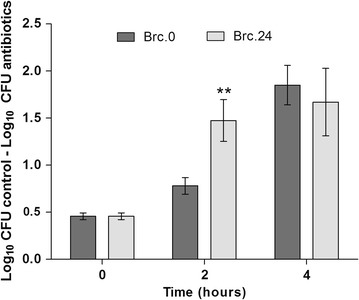
Brc susceptibility to vancomycin after transference to planktonic mode of growth. The total number of Brc obtained immediately after medium replacement (Brc.0) or after 24 h of growth in the presence of the originating biofilms (Brc.24) were determined by flow cytometry, and then adjusted to 1 × 10^7^ cells/mL in TSB_0.25 %G_. Thereafter, these cultures were grown in planktonic mode for up to 4 h. Every 2 h, an aliquot was collected, counted by flow cytometry and diluted down, to a concentration of 1 × 10^7^ cells/mL, in TSB_0.25 %G_ or TSB_0.25 %G_ with vancomycin and incubated, for 2 h, at 37 °C and 120 rpm. *Bars* represent the average ± standard deviation of three independent experiments. Statistical significance was determined using Unpaired *T* test (Two-tailed) with Welch’s correction. **P < 0.01

Bacterial cells within biofilms are known to be more tolerant to several families of antibiotics, as compared with their planktonic counterparts (Cerca et al. 2005a). In order to determine if Brc resemble biofilm or planktonic cells, the susceptibility of these three *S. epidermidis* populations to different classes of antibiotics, with distinct mechanisms of action, was evaluated. As expected, exponential phase planktonic cultures showed the highest susceptibility to vancomycin, tetracycline and rifampicin, while biofilm cells were the most tolerant of all (Fig. 5). Brc obtained immediately after medium replacement of the 24 h-old biofilms (Brc.0) had the same susceptibility profile as biofilm cells. While no differences were found in the case of vancomycin or rifampicin, the susceptibility of Brc to tetracycline was significantly reduced when compared with the other bacterial populations. The susceptibility of Brc.0 or Brc.24 to vancomycin was also addressed after growing these cells, for a few hours, in planktonic mode of growth. As shown in Fig. 6, both bacterial cultures regain susceptibility to vancomycin, although at slightly different rates, suggesting that Brc phenotype is transient.

### Biofilm-released cells present a distinct gene expression profile than planktonic and biofilm cells

**Fig. 7:**
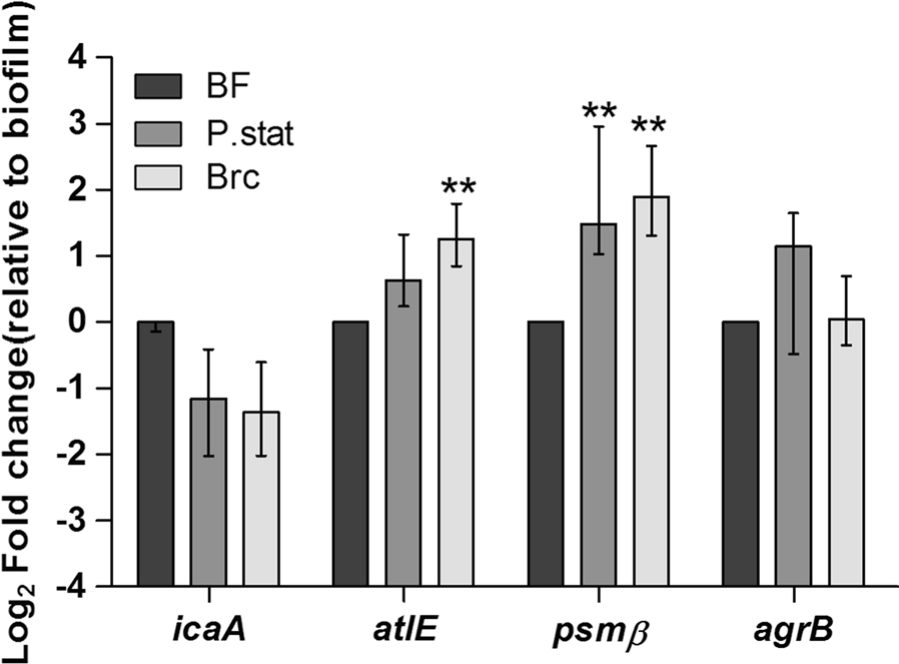
Gene expression profile of Brc and planktonic cells compared with biofilms. Quantitative PCR was performed in order to quantify the transcription level of genes involved in quorum sensing, biofilm accumulation or dispersion. *Bars* represent the median ± interquartile range of at least four independent experiments. Statistical significance was determined using Kruskal–Wallis and Dunn’s multiple comparison test. **P < 0.01

Since shear force-independent biofilm disassembly was observed, we characterized the three populations of cells regarding the transcription of genes known to be involved in quorum sensing (*agrB*), biofilm accumulation (*icaA*) and biofilm dispersion (*atlE*, *psmβ)* (Fig. 7). Overall, the profile of the Brc had more similarities to that of stationary planktonic bacteria than to that of biofilm cells, with the exception of the *atlE* transcripts, which were found to be significantly increased in Brc. Curiously, *agrB* transcription was highly variable and no significant changes were found among populations. Not surprisingly, biofilms had a higher transcription of *icaA* and lower of *psmβ*.

## Discussion

The release of cells from biofilms formed on the top of medical devices has been associated with the emergence of serious acute infections. However, despite the clinical relevance of these cells, little is known regarding their phenotype or interaction with the host immune system. Hence, more efforts towards the development of affordable models allowing the collection and posterior study of Brc cells are necessary. One of the most studied factors capable of initiating biofilm dispersion is the availability of essential nutrients (Hunt et al. 2004; Sauer et al. 2004). Glucose, an important carbon source for bacterial growth, is known to play a major role in *S. epidermidis* biofilm formation (Mack et al. 1992). Hence, we evaluated the influence of glucose in *S. epidermidis* biofilm dispersion. Interestingly, when no supplementary glucose was added to the culture medium, practically no differences were found between 24 and 48 h biofilms biomass, while with the highest glucose concentration tested, biofilm accumulation more than doubled, and disassembly was minimized. This was not surprising as Jäger and colleagues had previously shown that glucose-limiting conditions would result in a decrease in biofilm biomass over time (Jager et al. 2005), although in that study they did not address the cells in the biofilm bulk fluid. In addition, recently, our group has demonstrated that during the shift from biofilm to planktonic mode of growth important alterations in the expression of genes involved in glucose metabolism (*icaA* and *rpiA*) occurred having a significant impact on *S. epidermidis* physiological state (Cerca et al. 2014). Taken together, these data indicate that, depending on the concentration used, glucose can direct *S. epidermidis* biomass toward biofilm accumulation or disassembly. Importantly, this phenomenon was observed in six additional clinical and five commensal isolates, strengthening the transversal role of glucose in *S. epidermidis* biofilm accumulation/disassembly modulation. For further characterization of this model, we have selected TSB supplemented with 0.65 % of glucose since this is the most used concentration for biofilm studies.

Although we have shown that low glucose content and the presence of shear forces result in the disassembly of established biofilms, one possible criticism to this model is related to the difficulty in determining if the cells present in the biofilm bulk fluid were mainly originated from biofilm disassembly or proliferation of planktonic bacteria carried-over from the initial 24 h of growth. Since this may depend on the replacement of spent medium and the efficacy of the inherent washing steps, we became interested in assessing the impact of medium replacement on biofilm detachment. By washing the same biofilms sequentially, we demonstrated that not only the number of cells recovered in the washing suspension significantly decreased from the first to the third wash, but stayed essentially constant after that. As this step is highly influenced by the operator, this experiment was repeated by independent investigators and similar results were obtained. Importantly, the two washing steps performed before the addition of fresh medium removed practically all non-adherent cells (~99.01 to 99.99 %). Thus, it is reasonable to assume that the remaining cells (~1 × 10^5^ cells/mL) would not be the main source of the growth detected in the biofilm bulk fluid, since the concentration of cells in the bulk fluid was determined to be at least 5 × 10^7^ cells/mL (Fig. 3). Nevertheless, to properly address this question, the number of cells in the suspensions, in the presence or absence of the originating biofilms, was monitored up to 3 h. Our results showed that the cells obtained using this model were indeed originated from the biofilm attached to the bottom of the plate, as the number of cells in biofilm-dependent cultures increased more than 20-fold in the 3 h-period tested, while it only increased approximately 2-fold when growing in the absence of biofilms. Additionally, by performing the same experiment under static conditions (in the absence of shear forces), it was still possible to observe the release of cells from the biofilm as the number of cells in the biofilm-dependent cultures still increased 6-fold. This demonstrated that, despite the passive released of cells, an active mechanism was also taking place, although at much lesser extent. Taken together, these two experiments suggested that the proposed in vitro fed-batch disassembly model enables the collection of cells released from *S. epidermidis* biofilms by both passive and active mechanisms.

Finally, two sets of experiments were performed in order to provide further evidence of the origin of the cells in the biofilm bulk fluid. One of the major aspects differentiating biofilms from planktonic cultures is their characteristic tolerance to antibiotics and particular gene expression profile. Previously it was thought that soon after being released from a biofilm, bacteria would regain the characteristic planktonic cell’s sensitivity to antimicrobials (Chua et al. 2014; Lauderdale et al. 2010). However, opposing observations have been reported for different bacterial species (Liu et al. 2013; Rollet et al. 2009). Our results showed that *S. epidermidis* Brc were more tolerant than planktonic cells to the three classes of antibiotics tested. An identical tolerance profile was observed between the cells recently detached from the biofilm (Brc.0) and biofilms cells suggesting that these cells were originated from the established biofilm. Moreover, Brc retained their antimicrobial tolerance phenotype, despite being in the planktonic mode of growth. Curiously, after 12 h (Brc.12), a significant reduction in the susceptibility to tetracycline was observed. This effect was kept constant up to 24 h (Brc.24) after the initial disassembly. Interestingly, we also showed that if Brc are in the presence of the originating biofilm, they could maintain the high tolerance to antibiotics but, if centrifuged, suspended and transferred to an independent flask, they would eventually lose that tolerance, as observed for other bacterial species (Chua et al. 2014). This phenomenon is likely to be related to quorum-sensing regulation, which has been shown to be involved in tolerance to antibiotics (Singh and Ray 2014).

In addition to the antibiotic susceptibility, the quantification of gene expression identified additional particularities associated with Brc. Dai et al. showed previously that a specific group of *S. epidermidis* clinical isolates had enhanced biofilm dispersion and this is related to increased *atlE* activity (Dai et al. 2012). They proposed that biofilm dispersion was occurring, at least partially, due to autolysis of biofilm cells and could be mediated by the *agr* system. While we did not detect significant changes in *agrB* transcription levels, *atlE* transcription was significantly upregulated in Brc. Furthermore, in an earlier study, Yao et al. also characterized the transcriptome of stationary planktonic cells, biofilms and the cells in the biofilm bulk fluid (Yao et al. 2005). Similar to our study, they found that *psmB* was downregulated in biofilms. However, in their study, a significant difference between stationary planktonic and the biofilm bulk cells was detected, which was not observed in our study. Discrepancies in *agr* regulation have been reported in different strains (Stevens et al. 2009), but this is not surprising since several studies have linked *agr* to both biofilm accumulation and dispersion, as reviewed by Novick and Geisinger (2008).

Importantly, we have to acknowledge that one disadvantage of this fed-batch system for fundamental studies is that it’s not possible to physically separate the cells released by passive from the ones release by active mechanisms. However, this model is suitable for functional studies, as in vivo disassembly is likely to involve both dispersal and detachment mechanisms and, therefore, the population obtained in this model will more closely relate the population present in an in vivo situation of biofilm infection.
